# Characterization of Natural Compounds as Inhibitors of NS1 Endonuclease from Canine Parvovirus Type 2

**DOI:** 10.4014/jmb.2211.11040

**Published:** 2023-02-13

**Authors:** So-Hyung Kwak, Hayeong Kim, Hyeli Yun, Juho Lim, Dong-Hyun Kang, Doman Kim

**Affiliations:** 1 Department of Agricultural Biotechnology, College of Agriculture and Life Sciences, Seoul National University, Seoul 08826, Republic of Korea; 2 The Institute of Food Industrialization, Institutes of Green Bio Science and Technology, Seoul National University, Pyeongchang-gun, Gangwon-do 25354, Republic of Korea; 3 Graduate School of International Agricultural Technology, Seoul National University, Pyeongchang-gun, Gangwon-do 25354, Republic of Korea; 4 Department of Food and Animal Biotechnology, Department of Agricultural Biotechnology, Center for Food and Bioconvergence, and Research Institute for Agricultural and Life Sciences, Seoul National University, Seoul 08826, Republic of Korea; 5 Fervere Campus Corporation, Pyeongchang-gun, Gangwon-do 25354, Republic of Korea

**Keywords:** Canine parvovirus 2, curcuminoids, endonuclease, sesame cake, turmeric, yerba mate

## Abstract

Canine parvovirus type 2 (CPV-2) has high morbidity and mortality rates in canines. Nonstructural protein 1 (NS1) of CPV-2 has endonuclease activity, initiates viral DNA replication, and is highly conserved. Thus, it is a promising target for antiviral inhibitor development. We overexpressed a 41.9 kDa active recombinant endonuclease in *Escherichia coli* and designed a nicking assay using carboxyfluorescein and quencher-linked ssDNA as substrates. The optimal temperature and pH of the endonuclease were 37°C and pH 7, respectively. Curcumin, bisdemethoxycurcumin, demethoxycurcumin, linoleic acid, tannic acid, and α-tocopherol inhibited CPV-2 NS1 endonuclease with IC_50_ values of 0.29 to 8.03 μM. The extracted turmeric, yerba mate, and sesame cake suppressed CPV-2 NS1 endonuclease with IC_50_ values of 1.48, 7.09, and 52.67 μg/ml, respectively. The binding affinity between curcumin, the strongest inhibitor, and CPV-2 NS1 endonuclease by molecular docking was −6.4 kcal/mol. Curcumin inhibited CPV-2 NS1 endonuclease via numerous hydrophobic interactions and two hydrogen bonds with Lys97 and Pro111 in the allosteric site. These results suggest that adding curcuminoids, linoleic acid, tannic acid, α-tocopherol, extracted turmeric, sesame cake, and yerba to the diet could prevent CPV-2 infection.

## Introduction

Canine parvovirus type 2 (CPV-2) has high morbidity and mortality rates in untreated domestic and wild canine adults (10%), particularly puppies (91%), worldwide [1]. The symptoms of CPV-2 infection are acute hemorrhagic enteritis, myocarditis, vomiting, diarrhea, and dehydration [1]. Since 1978, CPV-2 has emerged in canids as a new host species, and likely originated from feline panleukopenia virus (FPV) [2, 3]. Its variants, such as CPV-2a, CPV-2b, and CPV-2, are undergoing evolution under immune pressure, mainly in capsid protein genes [2]. Continual development of vaccines against CPV-2, and persistent interference with the response to vaccination by maternal antibodies in the colostrum, hamper prevention of this infection [1]. Because canine parvovirus replicates via active mitosis of host cells, young puppies (from 6 weeks to 6 months age; particularly at < 12 weeks of age) are more prone to developing severe illnesses such as myocarditis [4]. The gap between passive immunity from the mother and active immunity via vaccination is a problem, as it coincides with the age at which puppies are most vulnerable to CPV-2 infection [5]. Thus, there is an urgent need for alternative prophylactics for CPV-2 infection.

CPV-2 (family *Parvoviridae*, subfamily *Parvovirinae*, genus *Protoparvovirus*, and species *Carnivore protoparvovirus* 1) has a single-stranded linear DNA genome of about 5.2 kb [6]. The CPV-2 virion is a nonenveloped icosahedral particle approximately 26 nm in diameter; it is composed of two nonstructural proteins (NS1 and NS2) encoded by one major open reading frame and three capsid proteins (VP1–VP3) encoded by a second major open reading frame and translated by alternative splicing of the same viral mRNAs [7, 8]. Among the three capsid proteins, VP2 is preferentially mutated and a major determinant of host range, antigenic properties, and receptor binding, making it an attractive target for CPV-2 vaccine development [9-11]. The NS1 sequence is conserved in CPV-2, 2a–2c [12, 13]. In CPV-2, NS1, a 76.7 kDa pleiotropic nuclear phosphoprotein belonging to the superfamily 3 (SF3) helicases, has a DNA-binding/endonuclease domain at the N-terminus, helicase domain at the center, and zinc-finger domain at the C-terminus [14]. NS1 controls DNA packaging into the capsid, cellular apoptosis, and binding of host proteins [15]. We focused on the N-terminus of NS1, which has a DNA replication origin-binding domain with endonuclease activity, and site-specifically nicks viral DNA to initiate its replication [15-17]. Therefore, inhibition of NS1 N-terminal endonuclease activity overcomes the problem of continuously mutating targets, such as VP2, and prevents viral proliferation by disrupting the initiation of viral genome replication at the S phase of the host cell cycle. The most vulnerable young puppies can be protected from CPV-2 infection by attenuating NS1 endonuclease activity, irrespective of the presence of maternal antibodies.

Diet is important for maintaining animal health, and the pet food market is growing as animal companionship becomes an ever-more-integral aspect of life [18]. Human–animal relationships influence mental, physical, and social health [19]. Increasing interest in animal welfare has led to demand for functional foods, and natural compounds are preferred by consumers for reasons of safety and nutritional adequacy [18]. However, the effects of natural compounds added to the diet on CPV-2 NS1 endonuclease have not been investigated. In this study, we investigated the inhibitory effects of 31 natural compounds on active recombinant CPV-2 NS1 endonuclease expressed in *E. coli* BL21 (De3) pLysS using a single-stranded DNA substrate (ssDNA). The inhibition mechanism of the best inhibitors on CPV-2 NS1 endonuclease was analyzed by molecular docking.

## Materials and Methods

### Design and Characterization of ssDNA Substrate

The fluorescence resonance energy transfer (FRET) substrate, which was composed of an oligonucleotide 5¢-TAA CCT TAC CAT AAG TA↓T CAA TCT GTC TTT-3¢ labeled with carboxyfluorescein (FAM) at the 5¢-terminus and black hole quencher 1 (BHQ1) at the 3¢-terminus as the energy transfer pair, was designed and synthesized by Bioneer Corp. (Korea) (Fig. S1).

### Preparation of CPV-2 NS1 Endonuclease

cDNA of the CPV-2 NS1 endonuclease gene was synthesized after codon optimization (Genscript, USA) based on the N-terminal amino acid sequence (amino acids 1–277 of the DNA-binding region) of CPV-2 NS1 endonuclease (GenBank Accession No. AAV36764.1) (Fig. S2). The CPV-2 NS1 endonuclease gene was imbedded into the pET-28a (+) vector (Novagen, Germany) with hexahistidine tags at the N- and C-termini. The plasmids were transformed into *E. coli* BL21 (De3) pLysS (Invitrogen, USA) for expression. The transformed cells were cultured in 1 L of LB containing 30 μg/ml kanamycin at 37°C until the OD_600_ reached 0.6. Next, protein expression was induced with 1 mM isopropyl β-D-1-thiogalactopyranoside (IPTG) at 24°C for 20 h. Cells were harvested by centrifugation at 12,000 ×*g* for 25 min and 4°C. The pellet was resuspended in 50 ml 50 mM Tris-HCl buffer (pH 7.0) containing 300 mM NaCl and disrupted by sonication. After centrifugation at 12,000 ×*g* for 30 min and 4°C, the supernatant was applied to a Ni-NTA agarose column (Qiagen, Germany) and washed with 50 mM Tris-HCl buffer (pH 8.0) containing 300 mM NaCl and 20 mM imidazole. CPV-2 NS1 endonuclease was eluted with 50 mM Tris-HCl buffer (pH 8.0) containing 300 mM NaCl, and 250 mM imidazole, and dialyzed against 50 mM Tris-HCl (pH 7.0) containing 300 mM NaCl at 4°C overnight. The purified protein was stored at −20°C until required.

### CPV-2 NS1 Endonuclease Activity

CPV-2 NS1 endonuclease activity was measured using an FRET substrate as described above. The reaction mixture was composed of 1.25 μg endonuclease and 0.6 μM FRET in 50 mM Tris-HCl buffer (pH 7.0). The reaction was run at 37°C with continuous monitoring of fluorescence for 10 min. Relative fluorescence units (RFUs) were measured using a SpectraMax M3 fluorescence plate reader (Molecular Devices, USA) with an excitation wavelength of 495 nm and an emission wavelength of 517 nm.

The effect of temperatures on CPV-2 NS1 endonuclease activity was evaluated by incubating 1.25 μg endonuclease and 0.6 μM FRET in 50 mM Tris-HCl buffer (pH 7.0) at 25°C, 37°C, 43°C, and 50°C for 10 min.

### Screening of Natural Compounds with Activity against CPV-2 NS1 Endonuclease

Thirty-one compounds, extracted sesame cake, turmeric, and yerba mate were tested for inhibitory effects against CPV-2 NS1 endonuclease. For screening, thirty-one compounds, turmeric, and yerba mate were dissolved in dimethyl sulfoxide (DMSO) to 5 mM or 10 mg/ml as stock solutions. Sesame cake extract (10 mg/ml) was solubilized in water. The reaction was initiated by adding 0.6 μM FRET substrate to a mixture of 50 μM compound and 1.25 μg endonuclease in 50 mM Tris-HCl buffer (pH 7.0) containing 300 mM NaCl. The reaction was run at 37°C for 3 min using a SpectraMax M3 fluorescence plate reader with an excitation wavelength of 495 nm and emission wavelength of 517 nm. Inhibitory activity was calculated using the following equation:

$${\text{Inhibition activity (\%) 100-[(S-S}}_{\text{0}}{\text{)÷(C-C}}_{\text{0}}\text{)]×100}$$

where C is RFU of the control (enzyme, buffer, and substrate) after a 3 min reaction, C_0_ is the RFU of the control at 0 min, S is the RFU of the sample (enzyme, buffer, inhibitor, and substrate) after 3 min, and S_0_ is the RFU of the sample at 0 min.

We determined the 50% inhibitory concentrations (IC_50_) of the six natural compounds, extracted turmeric, yerba mate, and sesame cake relative to the inhibitor-free control. The IC_50_ values of curcumin, and extracted sesame cake were calculated using a four-parameter logistic curve (4PL) using Prism v. 8 software (GraphPad Software Inc., USA). The IC_50_ values of tannic acid, demethoxycurcumin, bisdemethoxycurcumin, α-tocopherol, linoleic acid, yerba mate, and turmeric were calculated by simple linear regression using Prism v. 8 software.

### Modeling of CPV-2 NS1 Endonuclease and Molecular Docking Study

A CPV-2 NS1 endonuclease homology model was generated by homology modeling, based on the NS1 protein crystal structure of minute virus of mice (MVM) (PDB ID: 4pp4; 65.3% similarity) using MODELLER software (v. 10.2; Modelled, USA) [20]. The binding pocket of the endonuclease, together with its area and volume, was analyzed using the Computed Atlas of Surface Topography of Protein (CASTp) v. 3.0 server [21]. Molecular docking of CPV-2 NS1 endonuclease with curcumin was performed using AutoDock Vina [22, 23]. Docking files were prepared using AutoDockTools software [24]. The grid box, with a grid spacing of 0.375 Å and dimensions of 88 × 60 × 72 points in the *x*-, *y*-, and *z*-directions, respectively, was centered on the following coordinates: *x*, 5.541; *y*, 52.005; and *z*, 27.516. This was done to cover the active-site pocket and adjacent areas, based on the CASTp results. The best conformation, *i.e.*, that with the lowest root-mean-square deviation value, was selected to calculate the binding energy between CPV-2 NS1 endonuclease curcumin. The H-bonds and hydrophobic interactions between curcumin and CPV-2 NS1 endonuclease were analyzed using LigPlot software (v. 4.5.3)[25].

### Statistical Analysis

Experiments were carried out in triplicate and the results are means ± standard errors of the mean (SEMs). Statistical comparisons of IC_50_ values of six compounds (curcumin, bisdemethoxycurcumin, demethoxycurcumin, linoleic acid, tannic acid, and α-tocopherol) and the effect of temperature on endonuclease activity were determined by one-way analysis of variance (ANOVA) in SPSS software v. 26.0 (IMB Crop., USA). A value of *p* < 0.05 was taken to indicate statistical significance

## Results and Discussion

### Preparation of CPV-2 NS1 Endonuclease

**Fig. 1:**
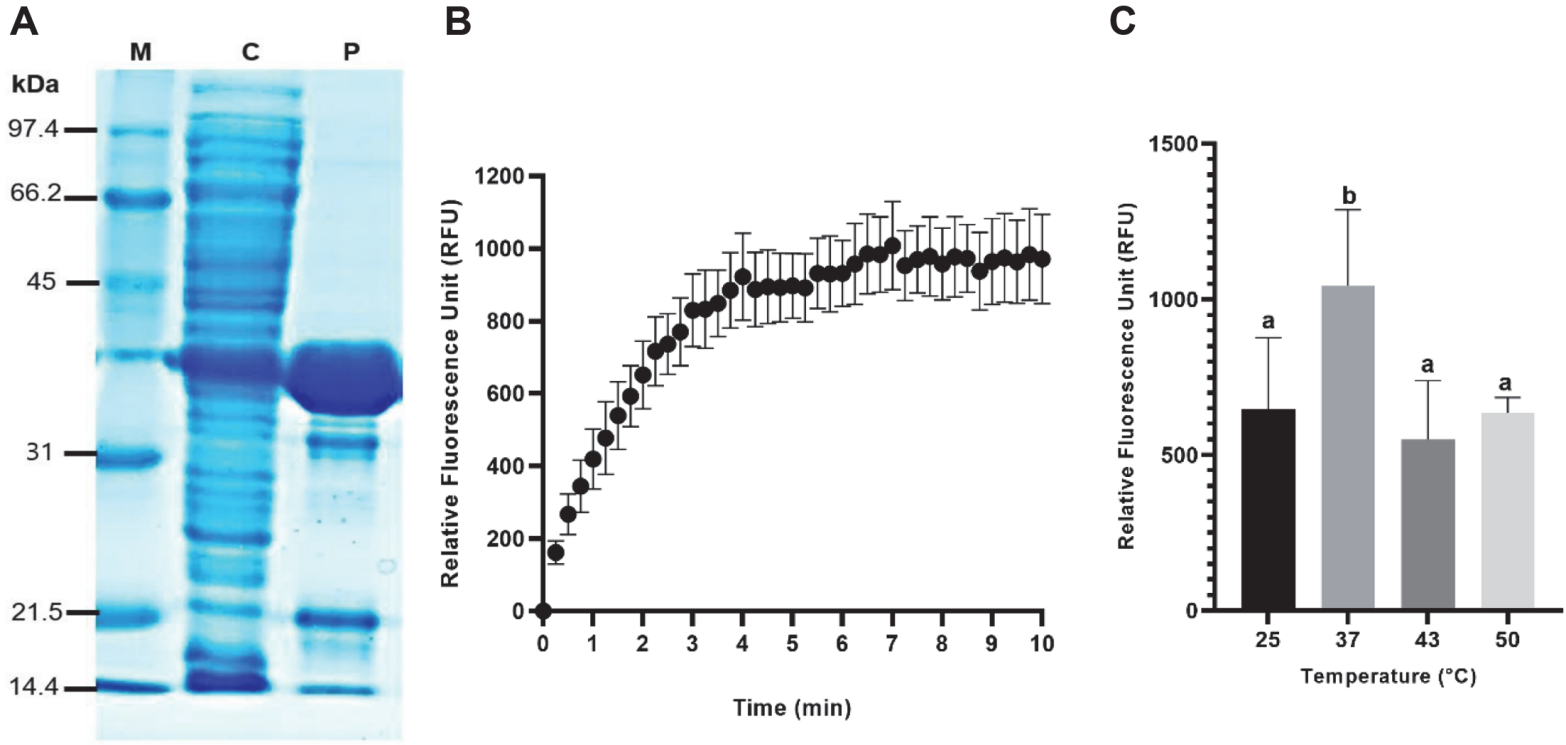
SDS-PAGE analysis of purified CPV-2 NS1 endonuclease (A), Relative Fluorescence Unit of singlestranded DNA by endonuclease reaction at 37°C and pH 7 (B), and the effect of temperature on endonuclease activity (C). Lane M, molecular markers; lane C, crude enzyme before purification; lane P, enzyme purified by Ni-agarose column chromatography. ^a, b^Significant differences (*p* < 0.05).

CPV-2 NS1 endonuclease was expressed (41.9 kDa) in *E. coli* BL21 (De3) pLysS at 24°C for 20 h using 1.0 mM IPTG and purified using a Ni-NTA agarose resin (Fig. 1A). The overall yield of CPV-2 NS1 endonuclease was 53.83% (Table 1).

The CPV-2 genome has inverted terminal repeats (ITRs) at both ends forming two hairpin structures, and the CPV-2 ssDNA genome uses the 3¢-end hairpin as a primer to lengthen viral ssDNA into the double-stranded DNA genome using host replication proteins [26]. In detail, a partially circular DNA genome forms by ligating the extended 3¢-end to the 5¢-end of the genome [26]. NS1 endonuclease nicks the terminal resolution site (trs) between the 5¢-end hairpins and newly elaborated viral DNA to expose a new 3¢ primer that triggers hairpin transfer (followed by strand displacement), via a modified rolling hairpin DNA replication mechanism in the nuclei of host cells [26, 27]. Also, CPV-2 NS1 nicks the site on the parental strand at a point opposite the original 3¢-terminus in a hairpin structure during DNA replication (Fig. S1) [26]. Therefore, 30 oligonucleotide substrates, including the nick site, were elaborated from the nucleotide sequence (GenBank Accession No. D26079.1)(Fig. S1). Purified CPV-2 NS1 endonuclease showed nicking activity with ssDNA substrate at 37°C and pH 7 (Fig. 1B). Although the expression of CPV-2 NS1 endonuclease in *E. coli* has been reported, most studies used recombinant CPV-2 NS1 endonuclease to prepare NS1 monoclonal antibodies [28, 29]. Thus, this is the first report of nicking activity between CPV-2 NS1 endonuclease and ssDNA. The effect of temperature on CPV-2 NS1 endonuclease activity using FRET as the substrate is shown in Fig. 1C. The highest CPV-2 NS1 endonuclease activity was obtained at 37°C. There was a nonsignificant difference in CPV-2 NS1 endonuclease activity at 25°C, 43°C, and 50°C.

### Screening of Natural Compounds with Activity against CPV-2 NS1 Endonuclease

**Fig. 2:**
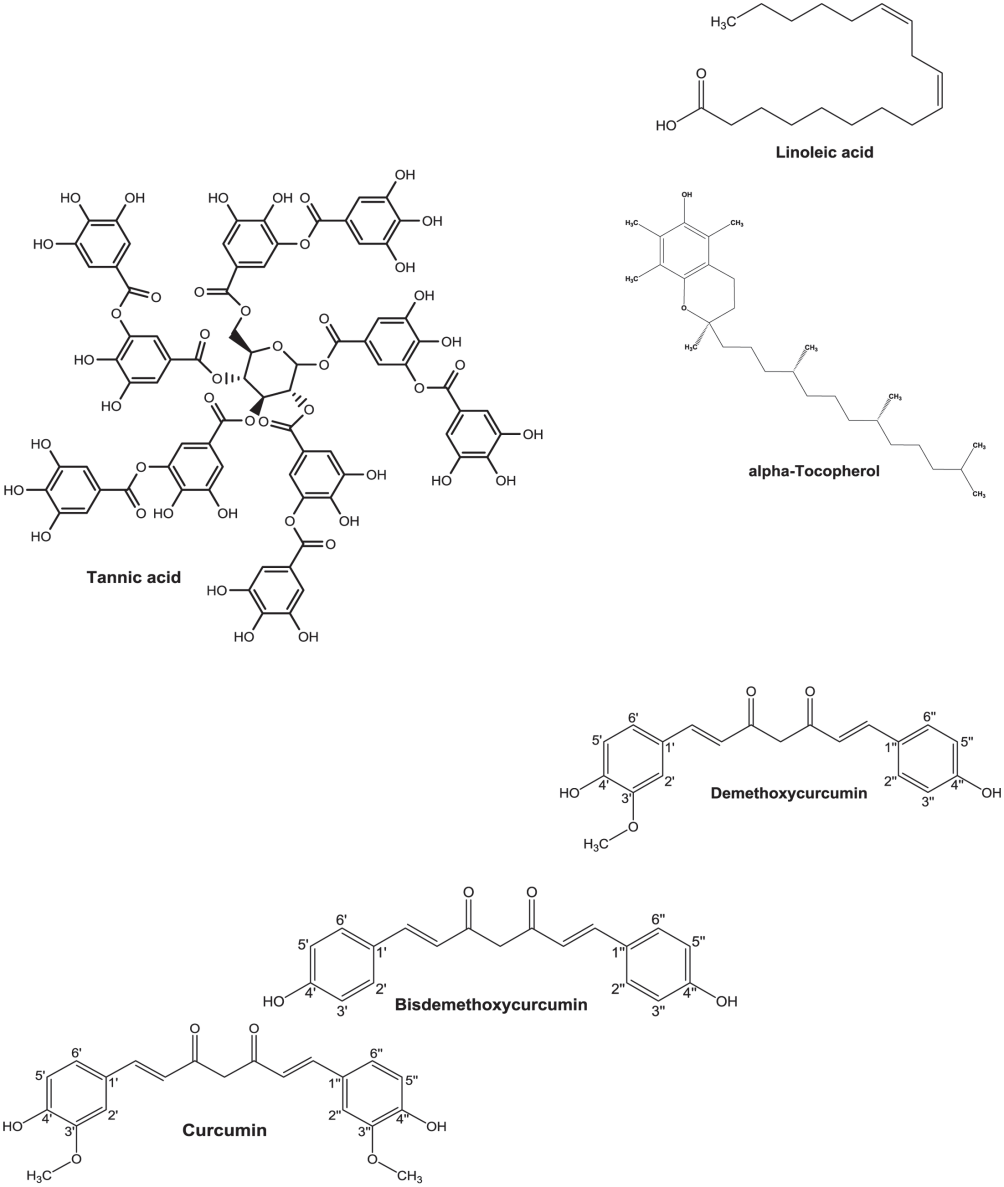
Chemical structures of tannic acid, α-tocopherol, linoleic acid, curcumin, demethoxycurcumin, and bisdemethoxycurcumin.

**Fig. 3:**
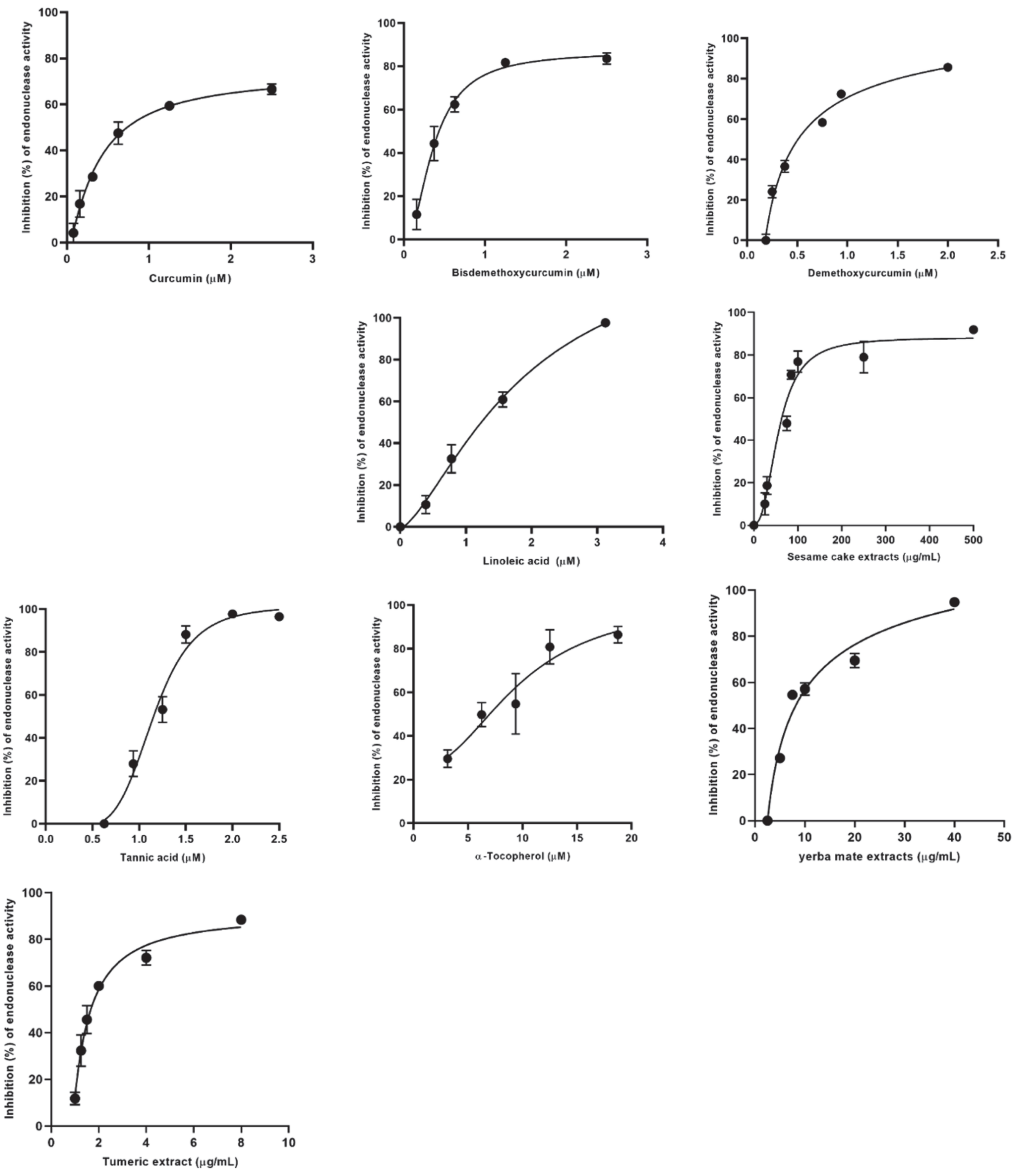
Dose-response curves of curcumin, bisdemethoxycurcumin, demethoxycurcumin, linoleic acid, tannic acid, α-tocopherol, extracted tumeric, extracted yerba mate, and extracted sesame cake. In FRET reaction, various concentrations of compound were added to the reaction mixture, which contained 0.6 μM FRET substrate and 1.25 μg of endonuclease in 50 mM Tris-HCl (pH 7.0) containing 300 mM NaCl. The reaction was run at 37°C for 3 min using a SpectraMax M3 fluorescence plate reader, with an excitation wavelength of 495 nm and emission wavelength of 517 nm. IC_50_ values were calculated using GraphPad Prism software. All experiments were performed in triplicate; data are means ± standard error of the mean (SEM).

The diet is an important part of dog care and crucial to maintaining health and the quality of life. There is much interest in canine products containing bioactive compounds to provide health benefits. Moreover, consumers accept natural compounds because they are considered safe. The inhibition by single compounds and extracts of CPV-2 NS1 endonuclease is shown in Table 2. At 50 μM, myricetin, fisetin, rutin, astragalin, naringenin, sesamin, sesamolin, piperine, L-ascorbic acid, mangiferin, and resveratrol did not inhibit CPV-2 NS1 endonuclease activity, whereas ferulic acid, chlorogenic acid, caffeic acid, ursolic acid, genistein, daidzein, epigallocatechin gallate, sesamol, caffeine, gallic acid, and L-carnitine inhibited it by <50% compared to the control. Oleanolic acid, quercetin, and vitamin K inhibited CPV-2 NS1 endonuclease activity by 51 ± 7%, 51 ± 3%, and 68 ± 15%, respectively. Curcumin, bisdemethoxycurcumin, demethoxycurcumin, tannic acid, linoleic acid, α-tocopherol, extracted sesame cake (100 μg/ml), turmeric (10 μg/ml), and yerba mate (40 μg/ml) inhibited CPV-2 NS1 endonuclease activity by >95%. The compounds and extracts that showed over 95% inhibitory activity against CPV-2 NS1 endonuclease were subjected to analysis of their IC_50_ values. The structures of curcumin, bisdemethoxycurcumin, demethoxycurcumin, linoleic acid, tannic acid, and α-tocopherol are shown in Fig. 2. The IC_50_ values of the compounds and extracts are shown in Table 3 and Fig. 3. For single compounds, the order of the inhibitory effect on CPV-2 NS1 endonuclease was as follows: α-tocopherol < tannic acid < linoleic acid < demethoxycurcumin < bisdemethoxycurcumin < curcumin. Among the 31 compounds of 16 groups, compounds in the tannoid, fatty acid, and diarylheptanoid groups showed the strongest inhibition of CPV-2 NS1 endonuclease.

Although curcumin had the strongest inhibitory activity against CPV-2 NS1 endonuclease, with an IC_50_ value of 0.29 ± 0.07 μM, followed by bisdemethoxycurcumin (IC_50_ value of 0.45 ± 0.05 μM), and demethoxycurcumin (IC_50_ value of 0.63 ± 0.01 μM), the inhibitory activities of curcumin and bisdemethoxycurcumin for CPV-2 NS1 were nonsignificant difference (Table 3). Interestingly, curcumin, demethoxycurcumin, and bisdemethoxycurcumin belong to the same diarylheptanoid group, but the magnitudes of their inhibitory effects on CPV-2 NS1 endonuclease differed. Curcumin, which has two methoxy groups at C3¢ and C3¢¢, showed 2.17-fold higher CPV-2 NS1 endonuclease inhibitory activity than demethoxycurcumin (one methoxy group at C3¢) (Table 3 and Fig. 2). Curcuminoids, natural polyphenol compounds derived from turmeric, contain 77% curcumin, 17%demethoxycurcumin, and 3% bisdemethoxycurcumin [30]. Extracted turmeric showed 100% inhibition of CPV-2 NS1 endonuclease activity at 10 μg/ml (Table 2) with an IC_50_ value of 1.48 ± 0.04 μg/ml (Table 3). Curcuminoids are used in feeds for animals such as lambs, chickens, dogs, and dairy sheep [31-34] due to their anti-inflammatory [30], antioxidant [35], antimicrobial [36], and anti-angiogenic [37] activities. Adding curcumin to the diet improved and preserved feed quality by increasing antioxidant levels and reducing lipoperoxidation [33, 34]. In the canine study, Campigotto *et al*. reported that a diet containing curcumin ameliorated the quality of feed and prolonged its preservation by increasing antioxidant levels and reducing lipoperoxidation [34]. Furthermore, it enhanced canine health by stimulating the antioxidant system and erythropoiesis [34].

Linoleic acid showed an inhibitory effect against CPV-2 NS1 endonuclease with IC_50_ values of 1.14 ± 0.04 μM (Table 3). Therefore, extracted sesame cake, which contains much linoleic acid, was tested for inhibitory effects against CPV-2 NS1 endonuclease. Extracted sesame cake (100 μg/ml) showed 100% inhibition of CPV-2 NS1 endonuclease activity with an IC_50_ value of 52.67 ± 2.08 μg/ml (Tables 2 and 3). Linoleic acid is the main fatty acid in sesame seed, sesame oil, and sesame cake (41.7%, 41.3%, and 42.4% of total fatty acids, respectively) [38, 39]. In addition to linoleic acid, α-tocopherol inhibited CPV-2 NS1 endonuclease with an IC_50_ value of 8.03 ± 0.80 μM (Table 3). The total vitamin E, composed of α-tocopherol, α-tocotrienol, and γ-tocopherol in sesame seed, sesame oil, and sesame cake was 432.0, 484.0, and 225.8 mg/kg, respectively [39]. To reduce the cost and waste from the sesame seed oil industry, sesame meal has been used as an alternative protein foodstuff to replace up to 15% of soybean meal in the diets of lactating Awassi ewes [40].

Tannic acid is a safe, complete feed for all animals up to the proposed maximum level of 15 mg/kg and is approved as a food additive by the United States Food and Drug Administration [41]. Yang *et al*. (2022) reported that dietary supplementation of tannic acid at 2.5 g/kg relieved environmental stress-induced diarrheal symptoms, inflammatory response, and oxidative stress in beagle dogs. Furthermore, tannic acid suppressed the growth of pathogenic bacteria and stimulated that of beneficial bacteria [42]. Tannic acid has antioxidant activity and inhibits lipid peroxidation [43]. In this study, tannic acid inhibited CPV-2 NS1 endonuclease activity with an IC_50_ value of 1.18 ± 0.10 μM (Table 3).

Yerba mate contains high levels of bioactive compounds, including chlorogenic acid, caffeine, theobromine, caffeic acid, 3, 4-dicaffeoylquinistudie 3, 5-dicaffeoylquinic acid, quercetin, kaempferol, rutin, oleanolic acid, ursolic acid, and tannic acid [44-47]. In this study, extracted yerba mate showed 100% inhibition of CPV-2 NS1 endonuclease activity at 40 μg/ml (Table 2) with an IC_50_ value of 7.09 ± 0.10 μg/ml (Table 3). Among its components, chlorogenic acid, caffeic acid, quercetin, tannic acid, oleanolic acid, and ursolic acid inhibited CPV-2 NS1 endonuclease activity by >29% at 50 μM (Table 2). Therefore, these compounds in yerba mate may contribute to its inhibitory effect against CPV-2 NS1 endonuclease activity.

Most of the compounds with high inhibitory activity against CPV-2 NS1 endonuclease were less- or insoluble in water [48]. Marchiori *et al*. (2019) tested the effects of free curcumin (30 mg/kg of feed), 3 mg nanoencapsulated curcumin/kg feed, and 10 mg nanoencapsulated curcumin/kg feed on bird eggs; egg production was higher under the free and 10 mg conditions than in controls. Egg weight and mass were higher under the free condition than in controls. Although the free curcumin treated group had higher antioxidant capacity than the control group, the treated group with 10 mg nanoencapsulated curcumin/kg feed showed the highest antioxidant activity, even though the curcumin concentration in feed was threefold lower than free curcumin [49]. Therefore, the inhibitors could be modified by, for instance, liposomes, phospholipids, nanoparticlese, and polymeric micelles, before being added to the canine diet to inhibit CPV-2 NS1 endonuclease. Further studies are needed.

### Modeling and Molecular Docking of CPV-2 Endonuclease with Curcumin

**Fig. 4:**
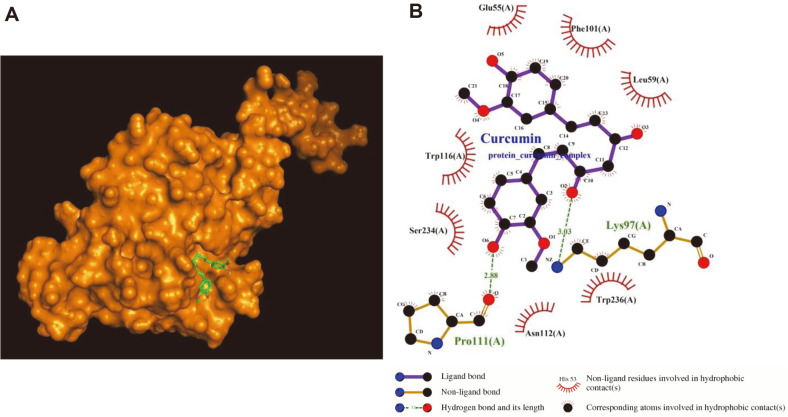
Molecular docking model and interaction between CPV-2 endonuclease curcumin. (**A**) Model of CPV-2 NS1 endonuclease with curcumin in the binding pocket. CPV-2 NS1 endonuclease homology model based on the NS1 protein crystal structure of MVM (orange, CPV-2 NS1; light green, curcumin). (**B**) Hydrophobic and H-bond interactions between curcumin and amino acid residues in the allosteric site of CPV-2 NS1 endonuclease. H-bond interactions, olive green dashed lines (red, oxygen; blue, nitrogen; black, carbon).

Among the 31 tested compounds, curcumin showed the strongest inhibitory effect against CPV-2 NS1 endonuclease; therefore, it was subjected to a molecular docking study. To investigate the interactions between CPV-2 NS1 endonuclease and curcumin, homology modelling of CPV-2 NS1 endonuclease based on the MVM NS1 protein crystal structure (PDB ID: 4pp4) was performed using MODELLER. As predicted by CASTp software, the binding pocket of CPV-2 NS1 endonuclease comprised 24 residues (Ser27, Phe28, Val29, Lys31, Glu121, Trp122, Gly123, Lys124, Asp125, Gln126, His129, His131, Leu133, Thr186, Ile187, Leu188, Thr189, Arg191, Val203, Met208, Tyr212, Phe213, Lys216, and Ser226). Molecular docking analysis using AutoDock Vina showed that the binding affinities of curcumin with CPV-2 NS1 endonuclease were −6.4 kcal/mol (Fig. 4A). Next, the hydrophobic and H-bond interactions between curcumin and amino acid residues in the allosteric site pocket of CPV-2 NS1 endonuclease were investigated using LigPlot software. Carbon and oxygen atoms of curcumin interacted hydrophobically with carbon, nitrogen, and oxygen atoms of Glu55, Leu59, Lys97, Phe101, Pro111, Asn112, Trp116, Ser234, and Trp236 of the endonuclease (Fig. 4B). Curcumin formed two H-bonds with residues in the allosteric site pocket of the endonuclease. The O^2^ atom of the hydroxyl group of the curcumin had one H-bond with the N atom of the side-chain amino group of Lys97 (at 3.03 Å). The O6 atom of the hydroxyl group of curcumin had one H-bond with the O atom of the carboxyl group of Pro111 (at 2.88 Å).

In conclusion, we expressed recombinant CPV-2 NS1 endonuclease and established a fluorescence endonuclease assay system to develop functional animal feed additives. Six bioactive compounds, including curcumin, bisdemethoxycurcumin, demethoxycurcumin, linoleic acid, tannic acid, and α-tocopherol, inhibited CPV-2 NS1 endonuclease, with IC_50_ values of 0.29–8.03 μM. Extracted turmeric, yerba mate, and sesame cake inhibited CPV-2 NS1 endonuclease with IC_50_ values of 1.48 ± 0.04, 7.09 ± 0.10, and 52.67 ± 2.08 μg/ml, respectively. The binding affinity of curcumin, the strongest inhibitor, with CPV-2 NS1 endonuclease was -6.4 kcal/mol and was mediated by numerous hydrophobic and two hydrogen interactions in the allosteric site. These results suggest that curcumin, bisdemethoxycurcumin, demethoxycurcumin, linoleic acid, tannic acid, α-tocopherol, extracted turmeric, yerba mate, and sesame cake could be added to the diet to prevent CPV-2 infection.

## Supplemental Materials

Supplementary data for this paper are available on-line only at http://jmb.or.kr.

## Figures and Tables

**Table 1:** Purification table of CPV-2 endonuclease expressed in *E. coli* BL21 (De3) pLysS.

Steps	Total volume (ml)	Protein (mg/ml)	Total protein (mg)	Total activity (FC)*	Specific activity (FC/mg)	Purification fold	Yield (%)
Culture supernatant	100	6.75	675	120 × 10^4^	1777.78	1	100
Ni-NTA purification	3	0.40	1.2	64.6 × 10^4^	538333	302.81	53.83

*FC (Fluorescence concentration) (FC=RFU·min^-1^ × D × V) (D: dilution factor, V: volume)

**Table 2:** Inhibitory activities of the natural compounds against CPV-2 endonuclease at 50 μM.

Group	Compound	Inhibition (%) at 50 μM
Diarylheptanoid	Curcumin	100
	Bisdemethoxycurcumin	100
	Demethoxycurcumin	100
Hydroxycinnamic acid	Ferulic acid	37 ± 5
	Chlorogenic acid	29 ± 4
	Caffeic acid	42 ± 8
Triterpenoid	Oleanolic acid	51 ± 7
	Ursolic acid	29 ± 5
Flavonol	Myricetin	0
	Fisetin	0
	Rutin	0
	Quercetin	51 ± 3
	Astragalin	0
Flavanone	Naringenin	0
Isoflavone	Genistein	45 ± 14
	Daidzein	22 ± 12
Flavan-3-ol	EGCG	49 ± 6
Furanoid lignan	(+)-Sesamin	0
Benzodioxole	Sesamolin	0
	Sesamol	9 ± 5
Alkaloid	Piperine	0
	Caffeine	7 ± 7
Tannoid	Tannic acid	100
Fatty acid	Linoleic acid	100
Tocopherol	α-Tocopherol (Vitamin E)	98 ± 3
	Vitamin K1	68 ± 15
Butenolide	L-Ascorbic acid	0
	Gallic acid	2 ± 2
Xanthonoid	Mangiferin	0
Stilbenoid	Resveratrol	0
	L-carnitine	29 ± 2
Mixture	Extracted sesame cake at 100 μg/ml	100
	Extracted tumeric at 10 μg/ml	95 ± 2
	Extracted yerba mate at 40 μg/ml	95

The reaction mixture contained 0.6 μM FRET substrate, 1.25 μg of enzyme, and 50 μM inhibitor in 50 mM Tris/HCl (pH 7.0).

The reaction was run at 37°C for 3 min.

Mean ± standard error of the mean (SEM)

**Table 3:** IC_50_ values of six compounds and extracted sesame cake against CPV-2 endonuclease.

Compounds	IC_50_ (μM or μg/ml)
Curcumin	0.29 ± 0.07^a^
Bisdemethoxycurcumin	0.45 ± 0.05^a^
Demethoxycurcumin	0.63 ± 0.01^ab^
Linoleic acid	1.14 ± 0.04^b^
Tannic acid	1.18 ± 0.10^b^
α-Tocopherol	8.03 ± 0.80^c^
Extracted sesame cake^1^	52.67 ± 2.08
Extracted tumeric^1^	1.48 ± 0.04
Extracted yerba mate^1^	7.09 ± 0.10

Mean ± standard error of the mean (SEM); ^a, b, c^Significant differences (*p* < 0.05).

^1^Represented for μg/ml
