# Supplementary material for: Characterization of Natural Compounds as Inhibitors of NS1 Endonuclease from Canine Parvovirus Type 2
Source: J Microbiol Biotechnol. 2023 Feb 13;33(6):788–96. doi: 10.4014/jmb.2211.11040 (PMC10331946; doi:10.4014/jmb.2211.11040)
Supplement: Supplementary file 1 [file jmb-33-6-788-supple.pdf]

J. Microbiol. Biotechnol.
https://doi.org/10.4014/jmb.2211.11040

Supplementary Table and Figures

Characterization of Natural Compounds as Inhibitors of NS1 Endonuclease from Canine Parvovirus Type 2

So-Hyung Kwak [1], Hayeong Kim[2], Hyeli Yun[3], Juho Lim[3], Dong-Hyun Kang [2, 4, *], and Doman Kim [2, 3, 5, *]

- **Table S1. Entire list of the natural compounds to inhibit CPV-2 endonuclease**
- **Fig. S1. Single stranded DNA of CPV-2 as substrates**
- **Fig. S2. Amino acid sequence of CPV-2 NS1 endonuclease**

Table S1. Entire list of the natural compounds to inhibit CPV-2 endonuclease

Group	Compound	Source	Country
**Diarylheptanoid**	Curcumin	TCI Chemical	Tokyo, Japan
	Bisdemethoxycurcumin	TCI Chemical	Tokyo, Japan
	Demethoxycurcumin	ChemFaces	Wuhan, China
**Hydroxycinnamic acid**	Ferulic acid	Fluka	Charlotte, NC, USA
	Chlorogenic acid	Sigma-Aldrich	St. Louis, MO, USA
	Caffeic acid	TCI Chemical	Tokyo, Japan
**Triterpenoid**	Oleanolic acid	TCI Chemical	Tokyo, Japan
	Ursolic acid	TCI Chemical	Tokyo, Japan
**Flavonol**	Myricetin	TCI Chemical	Tokyo, Japan
	Fisetin	TCI Chemical	Tokyo, Japan
	Rutin	Acros organics	Waltham, MA, USA
	Quercetin	Sigma-Aldrich	St. Louis, MO, USA
	Astragalin	Sigma-Aldrich	St. Louis, MO, USA
**Flavanone**	Naringenin	Sigma-Aldrich	St. Louis, MO, USA
**Isoflavone**	Genistein	Sigma-Aldrich	St. Louis, MO, USA

	Daidzein	Sigma-Aldrich	St. Louis, MO, USA
**Flavan-3-ol**	EGCG	Sigma-Aldrich	St. Louis, MO, USA
**Furanoid lignan**	(+)-Sesamin	TCI Chemical	Tokyo, Japan
**Benzodioxole**	Sesamolin	ChemFaces	Wuhan, Hubei, China
	Sesamol	TCI Chemical	Tokyo, Japan
**Alkaloid**	Piperine	TCI Chemical	Tokyo, Japan
	Caffeine	Sigma-Aldrich	St. Louis, MO, USA
**Tannoid**	Tannic acid	Sigma-Aldrich	St. Louis, MO, USA
**Fatty acid**	Linoleic acid	Sigma-Aldrich	St. Louis, MO, USA
**Tocopherol**	α-Tocopherol (Vitamin E)	Sigma-Aldrich	St. Louis, MO, USA
	Vitamin K1	Sigma-Aldrich	St. Louis, MO, USA
**Butenolide**	L-Ascorbic acid	Mallinckrodt	Surrey, United Kingdom
	Gallic acid	Sigma-Aldrich	St. Louis, MO, USA
**Xanthonoid**	Mangiferin	TCI Chemical	Tokyo, Japan

**Stilbenoid**	Resveratrol	TCI Chemical	Tokyo, Japan
	L-carnitine	TCI Chemical	Tokyo, Japan
**Mixture**	Sesame cake	Ottogi Sesame Mills	Eumseong-gun, Korea
	Tumeric	Ottogi Co., Ltd.	Eumseong-gun, Korea
	Yerba mate	Cheongmyeong Herb	Chungju-si, Korea

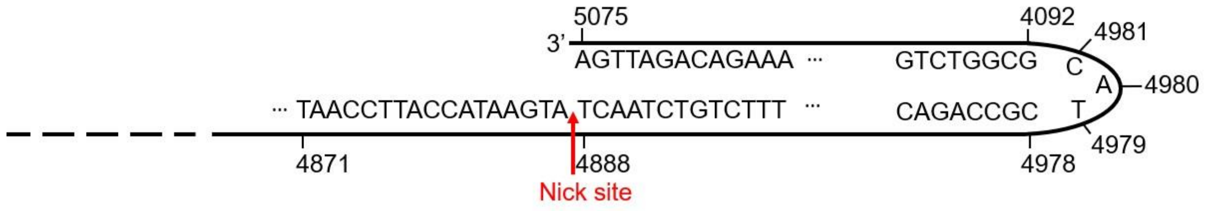

Fig. S1. Single stranded DNA of CPV-2 as substrates

MSGNQYTEEVMEGVNWLKKHAENEAFSFVFKCDNVQLNGKDVRWNNYTKPIQNE

ELTSLIRGAQTAMDQTEEEEMDWESEVDSLAKKQVQTFDALIKKCLFEVFVSKNIEP

NECVWFIQHEWGKDQGWHCHVLLHSKNLQQATGKWLRRQMNMYWSRWLVTLCS

VNLTPTEKIKLREIAEDSEWVTILTYRHKQTKKDYVKMVHFGNMIAYYFLTKKKIVH

MTKESGYFLSTDSGWKFNFMKYQDRQIVSTLYTEQMKPETVETTVTTAQETKRGRI

Fig. S2. Amino acid sequence of CPV-2 NS1 endonuclease
